# A Real-Time PCR Assay for the Quantification of *Plasmopara viticola* Oospores in Grapevine Leaves

**DOI:** 10.3389/fpls.2020.01202

**Published:** 2020-08-07

**Authors:** Melissa Si Ammour, Federica Bove, Silvia Laura Toffolatti, Vittorio Rossi

**Affiliations:** ^1^ Department of Sustainable Crop Production, DI.PRO.VE.S., Università Cattolica del Sacro Cuore, Piacenza, Italy; ^2^ Dipartimento di Scienze Agrarie e Ambientali - Produzione, Territorio e Agroenergia (DiSAA), Università degli Studi di Milano, Milano, Italy

**Keywords:** grapevine downy mildew, oospore density, qPCR, infestation level, overwintering inoculum, microscope counts

## Abstract

Grapevine downy mildew caused by *Plasmopara viticola* is one of the most important diseases in vineyards. Oospores that overwinter in the leaf litter above the soil are the sole source of inoculum for primary infections of *P. viticola*; in addition to triggering the first infections in the season, the oospores in leaf litter also contribute to disease development during the season. In the current study, a quantitative polymerase chain reaction (qPCR) method that was previously developed to detect *P. viticola* DNA in fresh grapevine leaves was assessed for its ability to quantify *P. viticola* oospores in diseased, senescent grapevine leaves. The qPCR assay was specific to *P. viticola* and sensitive to decreasing amounts of both genomic DNA and numbers of *P. viticola* oospores used to generate qPCR standard curves. When the qPCR assay was compared to microscope counts of oospores in leaves with different levels of *P. viticola* infestation, a strong linear relationship (R^2^ = 0.70) was obtained between the numbers of *P. viticola* oospores per gram of leaves as determined by qPCR vs. microscopic observation. Unlike microscopic observation, the qPCR assay was able to detect significant differences between leaf samples with a low level of oospore infestation (25% infested leaves and 75% non-infested leaves) vs. samples without infestation, and this ability was not influenced by the weight of the leaf sample. The results indicate that the qPCR method is sensitive and provides reliable estimates of the number of *P. viticola* oospores in grapevine leaves. Additional research is needed to determine whether the qPCR method is useful for quantifying *P. viticola* oospores in grapevine leaf litter.

## Introduction

Diseases represent a constant threat to grapevine production and may cause considerable yield and economic losses. Grapevine downy mildew (DM), which is caused by the oomycete *Plasmopara viticola* (Berk et Curt.) Berlese and de Toni, is an important disease in vineyards located in areas with frequent rain and planted with susceptible cultivars of *Vitis vinifera* (Lafon and Clerjeau, 1988).

*P. viticola* has dimorphic reproductive forms, i.e., sexual and asexual forms, which are responsible for primary and secondary DM infections, respectively. Oospores represent the sexual stage of *P. viticola* and are formed after the fusion of an antheridium and an oogonium in the affected leaf tissue from mid-summer to autumn (Burruano, 2000; Rossi et al., 2013). The formation of oospores does not require particular temperatures but seems to be favoured by dry conditions (which impede asexual sporulation) or by leaf senescence (Gessler et al., 2011). Oospores overwinter in the leaf litter above the soil surface (Kennelly et al., 2007; Rossi et al., 2013). During winter, oospores reach morphological maturity, i.e., the oospore wall becomes thick, the nuclei fuse, an ooplast is formed, and large lipid globules separate into smaller ones (Vercesi et al., 1999). Germination of morphologically mature oospores is prevented by dormancy (Galet, 1977; Rossi and Caffi, 2007), a process regulated by the environment, nutrient permeability, and endogenous inhibitors. When the dormancy is broken, oospores are considered physiologically mature and are able to germinate under favorable environmental conditions (Rossi and Caffi, 2007). Oospore germination ends with the formation of a macrosporangium containing zoospores (Galbiati and Longhin, 1984). The germination of oospores requires a minimum temperature of 12–13°C (the optimum is between 20 and 24°C) (Laviola et al., 1986; Gessler et al., 2011), and also requires the moistening of the leaf litter by rainfall or water flux from the atmosphere (Arens, 1929; Rossi et al., 2008a; Rossi et al., 2013). Dry conditions prolong the dormant period and may damage oospores if protracted (Arens, 1929; Gessler et al., 2011). Some oospores remain dormant but viable for an entire season or even for several years (Kennelly et al., 2007; Caffi et al., 2011).

Oospores are the sole source of inoculum for primary infections of *P. viticola*, and were long considered to play a role only in triggering the epidemic in the early grapevine season; the subsequent explosive increase of the disease was attributed to asexual multiplication and secondary infections (Blaeser and Weltzien, 1979; Lalancette et al., 1988; Lafon and Clerjeau, 1988). The use of DNA microsatellites, which enables the identification of genotypes causing single DM leaf lesions, showed that new *P. viticola* genotypes enter into the epidemic during most of the grape-growing season, indicating that oospores continue to germinate throughout the season, and that the primary inoculum not only triggers epidemics but contributes to their progress (Kump et al., 1998; Gobbin et al., 2003; Rumbou and Gessler, 2004; Gobbin et al., 2005; Gobbin et al., 2006). Oospores constitute a very large and diverse inoculum pool, leading to a pathogen population with high genotypic diversity. This diversity allows the pathogen to adapt to several microclimates and various host varieties (Rossi et al., 2013).

The detection of *P. viticola* oospores in grape leaf tissue has been mainly based on two methods: i) direct microscopic observations after leaf clearing and staining (Burruano, 2000; Díez-Navajas et al., 2007; Taylor, 2018) or after filtration of the leaf tissue (Vercesi et al., 2000; Toffolatti et al., 2007); and ii) indirect estimation *via* a “floating grape leaf disc” bioassay (Hill, 1998). In the latter assay, leaf fragments containing oospores are immersed in water in the presence of non-infested floating leaf discs so that the zoospores originating from the oospores swim to the stomata of the discs and cause infection, the severity of which is proportional to the oospore number. These methods have been used to study oospore formation and germination processes (Lehoczky, 1965; Vercesi et al., 2002; Vercesi et al., 2010), mating types (Wong et al., 2001; Taylor, 2018), fungicide resistance in oospores (Bissbort et al., 1997; Wong and Wilcox, 2001; Gisi et al., 2007; Toffolatti et al., 2007; Toffolatti et al., 2011; Toffolatti et al., 2018), and oospore production in partially resistant grapevine cultivars (Brown et al., 1999; Delbac et al., 2018). The methods have also been used to assess the effect of environmental conditions on oospore maturation and germination dynamics in vineyards (Hill, 2001; Pertot and Zulini, 2003; Toffolatti et al., 2004; Rossi et al., 2008a; Caffi et al., 2009).

No studies have been conducted with the goal of enumerating the oospores in leaf litter in order to assess of the *P. viticola* inoculum potential in a vineyard. A possible reason is that both microscopy and leaf disc bioassays are too expensive and time-consuming for assessing the high numbers of oospores (as many as several hundred per g of leaf residue) that can be present in the leaf litter. Molecular methods that have been used for the quantification of the inoculum in other pathosystems (Pavón et al., 2008; Li et al., 2010; Hussain et al., 2014; Van der Heyden et al., 2019) could be potentially used for *P. viticola*. Although polymerase chain reaction (PCR) and real-time PCR (Valsesia et al., 2005; Gindro et al., 2014) have been developed for the detection and quantification of *P. viticola* in fresh grape leaves, molecular methods have not been developed for the detection and quantification of oospores.

The objective of this study was to determine whether a quantitative real-time PCR (qPCR) assay previously developed for the detection of *P. viticola* DNA in fresh leaves (Valsesia et al., 2005) could be used to quantify *P. viticola* oospores in diseased, senescent grapevine leaves.

## Materials and Methods

### Plant and *P. viticola* Material

In 2018, *P. viticola* sporangia and oospores were collected from vine plots that had not been treated with fungicides and that were located in an experimental vineyard (cv. Merlot) on the campus of Università Cattolica del Sacro Cuore (UCSC) in Piacenza (Italy).

In late May of 2018, sporangia of *P. viticola* were collected from leaves showing typical DM lesions (oil spots) with fresh sporulation on the abaxial surface of the leaf blade. A sterile cotton swab was used to gently remove the sporangia, which were suspended in sterile-distilled water and examined with a light microscope (40× magnification). After the sporangial suspension was centrifugated at 14,000 rpm for 10 min, the aqueous phase was discarded, and the sporangia in the pellet were stored at −20°C until they were used for extraction of genomic DNA.

**Figure 1:**
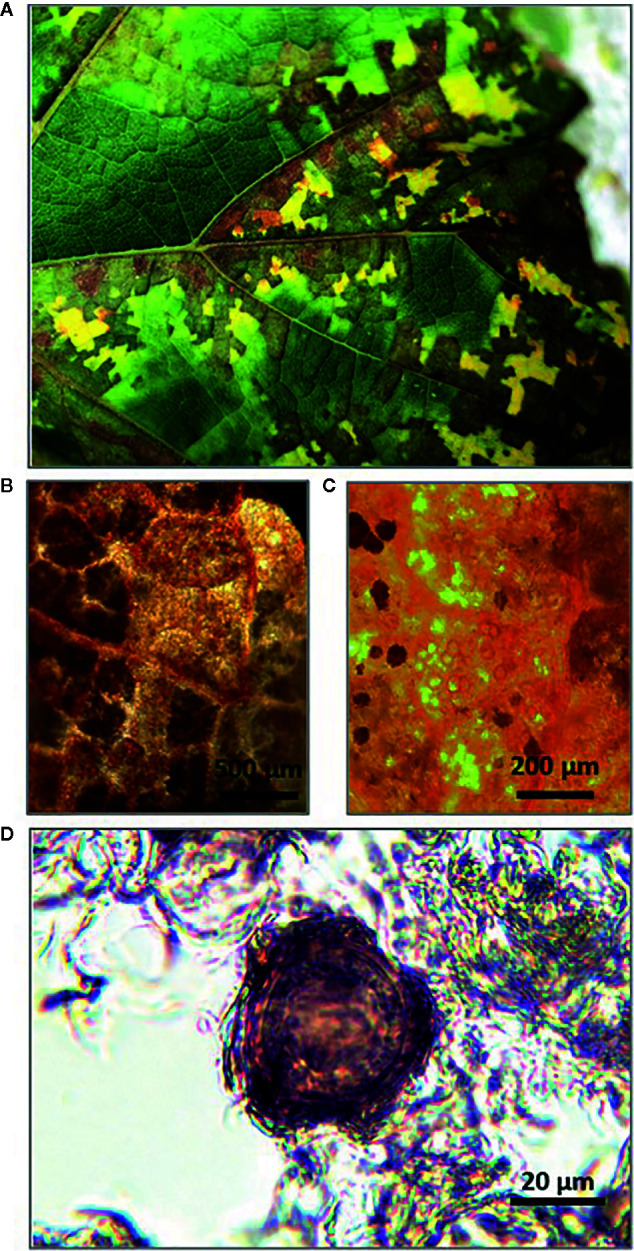
Senescent grape leaves showing typical downy mildew mosaic-like symptoms collected at the end of grapevine growing season **(A)**; Stereomicroscope observation of *Plasmopara viticola* oospores inside bleached leaf pieces at 10× **(B)** and 20× **(C)**; Light microscope observation of *P. viticola* oospore in suspension at 40× **(D)**.

In late September of 2018, leaves showing typical DM mosaic-like symptoms were collected from vines in the experimental vineyard (cv. Merlot) (**Figure 1A**). Although still attached to the vines at the time of collection, the leaves were diseased and senescent and would have soon fallen to the soil surface. The leaf petioles were removed and the leaf blades were wrapped in moistened blotting paper to prevent desiccation, placed in polyethylene bags, and stored at 4°C. To confirm the presence of *P. viticola* oospores in the freshly collected leaves, leaf pieces (1–2 cm^2^) with typical mosaic-like lesions were immersed in an acetic acid-ethanol (1/3 v/v) solution overnight at room temperature. The bleached leaf pieces were rinsed in distilled water and examined by light microscopy at 10–20× magnification (**Figures 1B, C**). Oospore suspensions were obtained as described by Vercesi et al. (2000), with a few modifications. Leaf pieces (approximately 1–2 cm^2^) were cut from the leaf blades with mosaic-like symptoms, weighed with an analytical balance (RADWAG PS45000/C2. Radom, Poland), and then finely ground in a mortar containing 20 mL of sterile-distilled water. The homogenate was passed through a series of three nylon filters with pore sizes of 100, 75, and 45 μm by thorough washing with sterile-distilled water. The material retained on the 45-μm mesh filter, which was presumed to contain the oospores based on oospore size, was resuspended in 15 mL of sterile-distilled water. The collected *P. viticola* oospores were counted with a hemacytometer at 40× (**Figure 1D**), and the counts were expressed as numbers of oospores per milliliter of suspension and per gram of leaf piece. The latter weight refers to leaves wrapped in blotting paper, for which 1 g of wrapped leaf = 1.3 g of fresh leaf = 0.34 g of dry leaf (i.e., after drying at 65°C for 24 h).

Non-infested, senescent leaves were also used, and these were collected from potted grape plants (cv. Merlot) that were growing in isolation in a greenhouse at the University campus.

### Real-Time qPCR

#### DNA Extraction Method

Genomic DNA was obtained from *P. viticola* sporangia suspensions, oospore suspensions, and oospore-containing leaf pieces. The DNA was extracted as described by Toffolatti et al. (2007) with minor modifications in sample preparation; a 1-mL volume of each sporangia and oospore suspension, prepared as described above, was centrifuged at 14000 rpm for 10 min, and the aqueous phase was discarded. The pellets of sporangia and oospores as well as leaf pieces containing oospores (100 mg) were placed in 2-mL microcentrifuge tubes containing 500 µL of cetyl trimethylammonium bromide (CTAB) extraction buffer (2% CTAB, 100 mM Tris-HCl pH 8.0, 20 mM ethylenediaminetetraacetic acid [EDTA], 1.4 M NaCl, and 1% polyvinylpyrrolidone [PVP]), 100 mg of glass sand (425–600 µm diameter), and two glass beads (5 mm diameter). The sporangia, oospores, or leaf pieces in the tubes were then ground for 1 min at 30 cycles/s with a TissueLyser II (Qiagen, Milano). The tubes were subsequently placed in a heat block at 65°C for 90 min. DNA was purified with chloroform-isoamyl alcohol (24:1) (v:v), precipitated with isopropanol, and resuspended in 40 µL of sterile ultrapure water. The yield and purity of the extracted DNA were determined using a NanoPhotometer^®^ N60 (Implen GmbH, München).

#### qPCR Assay

The qPCR assay was based on two specific primers and a hydrolysis probe (Giop) designed to target the internal transcribed spacer 1 (ITS 1) -5.8S rDNA of *P. viticola* (Valsesia et al., 2005), with the fluorescent reporter FAM (6-carboxyfluorescein) as a substitute for VIC reporter dye. The primer sequences were as follows: Giop F: 5′-TCC TGC AAT TCG CAT TAC GT-3′; Giop R: 5′-GGT TGC AGC TAA TGG ATT CCT A-3′; Giop P: 5′-6-FAM-TCG CAG TTC GCA GCG TTC TTC A-BHQ-1-3′. Reaction mixtures contained 1× Luna Universal Probe qPCR Master Mix (New England Biolabs, Ipswich), 250 nM of probe GiopP, 700 nM of each primer (GiopF/R), and 2 µL of DNA template in a final volume of 10 µL. An Applied Biosystems StepOnePlus™ System (Thermo Fisher Scientific Inc., Waltham) was used, and reaction conditions consisted of an initial incubation at 95°C for 1 min followed by 40 cycles of 95°C for 15 s and 60°C for 30 s.

#### Specificity

The specificity of the qPCR assay for *P. viticola* was determined in a test that included fungi and other oomycetes frequently found in grapevines (**Table 1**); these species were not previously assessed by Valsesia et al. (2005). The fungal strains were obtained from the culture collection of the Department of Sustainable Crop Production of the UCSC, Piacenza (Italy). *Erysiphe necator* Schwein. isolates were collected in the field in 2017 and 2018 and were maintained in the greenhouse on inoculated grape plants (cv. Merlot). Except in the case of *P. viticola* and *E. necator*, genomic DNA was obtained from 100 mg of fresh mycelium (obtained by scrapping the surface of 10-day-old colonies growing on potato dextrose agar, PDA). Genomic DNA was obtained from sporangial suspensions in the case of *P. viticola* and from leaf discs with sporulating powdery mildew colonies (100 mg of leaf material) in the case of *E. necator*. The DNA extraction method was previously described by Si Ammour et al. (2019).

**Table 1:** List of fungi, oomycetes, and plants used for testing the specificity of a real-time qPCR assay targeting *Plasmopara viticola* DNA, and the corresponding results.

**Genus and species**	**Isolate code^a^**	**qPCR result**
*Alternaria alternata*	5	−^b^
*Alternaria* sp.	23	−
*Aspergillus flavus*	4	−
*Aspergillus niger*	3	−
*Botrytis cinerea*	351V	−
*Botrytis cinerea*	213 T	−
*Guignardia bidwelii*	Q15	−
*Monilia laxa*	11	−
*Penicillium* sp.	2	−
*Phomopsis viticola*	Ph-1	−
*Rhizopus* sp.	26	−
*Sclerotinia sclerotiorum*	22	−
*Stemphylium* sp.	14	−
*Erysiphe necator*	FP^c^ 2017 and FP 2018	−
*Vitis vinifera*	N/A	−
*Plasmopara viticola*	FP 2017 and FP 2018	+

^a^Codes refer to the culture collection of the Department of Sustainable Crop Production of the University of Piacenza (Italy); ^b^ + indicates amplified, and − indicates not amplified; ^c^ FP indicates a field population with the year of collection.

#### qPCR Standard Curves

The analytical sensitivity of the assay was assessed following an absolute quantification approach. Standard curves of the Giop assay were obtained using genomic DNA of *P. viticola* as template in a 10-fold dilution series (from 1 to 10–5 ng), and DNA from oospores suspensions as template in a 10-fold dilution series (from 12,000 to 1 oospore/mL, equivalent to 6,000 to 0.5 oospores/g of leaf sample). Real-time qPCR assays were carried out twice, and for each DNA template, each dilution was replicated three times. A water control was included in triplicate in each assay. Standard curves of the qPCR assay were produced by linear regression, and the coefficient of determination (R^2^) was calculated. The amplification efficiency (E) was determined from the slope of the standard curves (Bustin et al., 2009).

### Microscope Counts vs. qPCR Assay

In experiment 1, five leaf samples with different levels of *P. viticola* infestation were prepared by mixing DM-infested and non-infested leaf pieces, described above, in the following proportions: 0 g infested/100 g non-infested (infestation 0%), 25/75 (25%), 50/50 (50%), 75/25 (75%), and 100/0. In experiment 2, leaf samples were prepared that consisted of 1, 2, or 3 g of DM-infested leaves.

Samples of both experiments were managed as described earlier, and numbers of oospores were determined by the Giop qPCR assay in three biological replicates in each assay and by microscope counts of oospore suspensions in four replicate samples for each infestation level in experiment 1 or in two replicate samples for each sample weight in experiment 2. Six replicate microscopic counts were carried out for each biological replicate sample in each experiment. The two experiments were performed twice.

### Data Analysis

All statistical analyses were carried out using SPSS (version 24; IBM SPSS Statistics, IBM Corp., USA). An analysis of variance (ANOVA) was used to test differences among leaf samples in the two experiments. Before ANOVA, numbers of oospores per g of leaf were transformed using the decimal logarithm function to make variances homogeneous. A post-hoc comparison of means was conducted by using the Student-Newman-Keuls test at P<0.05.

## Results

### qPCR Specificity and Standard Curves

In the specificity test for *P. viticola*, the Giop probe/primer set did not amplify the purified DNA of non-target organisms but did amplify the purified DNA of *P. viticola* (**Table 1**). The Giop standard curve generated by a 10-fold serial dilution of *P. viticola* purified DNA obtained from sporangia suspensions was linear, with an efficiency of 110% and an R^2^ (the coefficient of determination) of 0.99 (**Table 2**, eq. 1). Similarly, the oospore standard curves generated by five-fold serial dilutions of extracted oospore DNA were linear, with an efficiency of 121% and an R^2^ of 0.99 (**Table 2**, eq. 2). The Giop assay was able to amplify the lowest tested concentration of *P. viticola* purified DNA, which was 10^−5^ ng, and a *P. viticola* oospore concentration as low as 1 oospore/mL (not shown). A third linear regression derived from the superposition of the two previous regressions was calculated for the relationship between DNA quantity (ng) and the number of oospores in a Cq range from 18 to 37, with an R^2^ of 0.99 (**Table 2**, eq. 3). Based on this equation, the oospore number increased linearly with the quantity of DNA in the leaf sample.

**Table 2:** Linear regressions, coefficients of determination (R^2^), and reaction efficiencies (E, %) for the qPCR standard curves used for the quantification of *Plasmopara viticola* oospores; Cq is the qPCR quantification cycle.

**Equation number**	**Linear equations^a^**	**R^2^**	**E%**
**1**	Cq = 21.60 − 3.08× log_10_ (DNA)	0.99	110
**2**	Cq = 36.262 − 2.903× log_10_ (est)	0.99	121
**3**	N oosp/g = 113,966× (DNA)	0.99	–

^a^Linear equations Y = a+bX: In equation 1, Y refers to the Cq value, and X refers to the log_10_ transformed DNA concentration (ng). In equation 2, Y refers to the Cq value, and X refers to the log_10_ transformed estimated (est) number of oospores per gram of leaf. In equation 3, Y refers to the number of oospores per gram of leaf (N oosp/g) and X refers to the DNA concentration (ng).

### Microscope Counts vs. qPCR Assay

In experiment 1, oospore numbers were estimated by microscope counts and by the qPCR assay in samples with the following percentages of *P. viticola*-infested leaves: 0, 25, 50, 75, and 100%. The ANOVA revealed significant differences among infestation levels, but the qPCR assay provided more consistent differences than the microscope counts, as indicated by the post-hoc test (**Table 3**). For instance, the post-hoc test for microscope counts revealed no significant difference between the number of oospores in the 25% and 0% infection level, which was not the case for the qPCR assay. The standard errors for the oospore estimation were greater with microscope counts than with qPCR estimation, especially for the 25 and 50% infestation levels, i.e., the variability among replicates was greater for the microscope counts than for the qPCR estimates (**Table 3**).

**Table 3:** Numbers of oospores per gram of leaf estimated using two methods (microscope count and qPCR) in grape leaves with different levels of infestation by *Plasmopara viticola* (experiment 1) and with different sample weights of infested leaves (experiment 2).

Experiment		Microscope count		Estimation by qPCR	
1	Infestation level (%)				
	0	0.000 ± 0.000	a	0.000 ± 0.000	a
	25	1.525 ± 0.881	ab	3.282 ± 0.229	b
	50	2.419 ± 0.811	bc	3.620 ± 0.280	bc
	75	3.805 ± 0.045	c	3.907 ± 0.270	cd
	100	3.861 ± 0.054	c	4.252 ± 0.230	d
	P	0.001		<0.001	
2	Sample weight (g)				
	1	3.815 ± 0.060	a	4.290 ± 0.061	a
	2	3.477 ± 0.103	b	4.194 ± 0.252	a
	3	3.525 ± 0.025	b	3.723 ± 0.139	a
	P	0.002		0.094	

Values are log_10_ means and standard errors of 4 and 12 replicates for experiment 1 and 2, respectively. P values indicate the significance level from ANOVA; letters indicate differences among means (P < 0.05) based on the Student-Newman-Keuls test at P = 0.05.

In experiment 2, the number of oospores per g of leaf was determined by microscope counts and by the qPCR assay in three sub-samples of different weights (1, 2, and 3 g); the sub-samples were obtained from the same infested leaf sample, with the expectation that the sub-sample weight would not influence the number of oospores detected per g of leaf. The ANOVA revealed significant differences between sample weights for microscope counts but not for qPCR (**Table 3**), showing that the former method was affected by the leaf sample weight while the latter was not.

**Figure 2:**
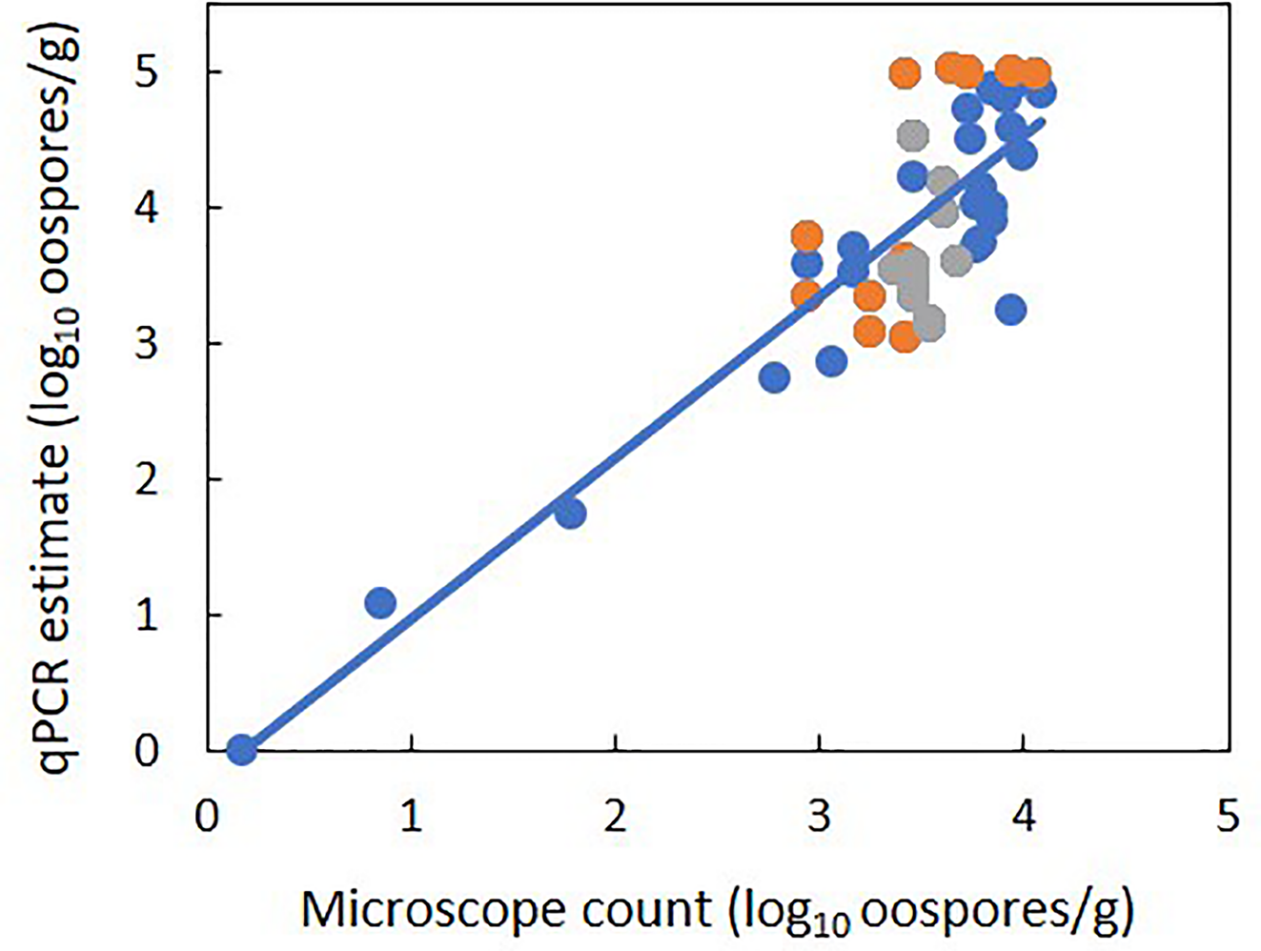
Relationship between estimates of the number of *Plasmopara viticola* oospores per gram of leaf (log_10_ transformed) based on the real-time qPCR assay and on microscope counts. Blue and orange dots represent mean values from experiment 1 and 2, respectively. Experiment 1 had five levels of *P. viticola* infested leaves (0, 25, 50, 75, and 100%) with a constant sample weight of 1 g. Experiment 2 had three samples weights of infested leaves: 1, 2, and 3 g. The line represents linear regression 1 in **Table 4**.

There was a linear relationship between the number of *P. viticola* oospores per gram of leaf (log10 transformed) estimated by qPCR and enumerated with the microscope, with R^2^ = 0.70 (**Figure 2**, **Table 4** eq. 1). In this linear equation, the intercept (a=−0.147 ± 0.362) and slope (b=1.157 ± 0.100) were not significantly different from zero (P=0.686) and one (P=0.134), respectively (**Figure 2**), indicating that the qPCR did not estimate the presence of oospores when there were no oospores in the leaf sample, and that the number of oospores detected by qPCR was equal to the number detected by microscopic observation. The relationship between the number of *P. viticola* oospores per gram of leaf (log10 transformed) estimated by qPCR and the Cq value is described by a logistic equation with a R^2^ of 0.87 (**Table 4** eq. 2).

**Table 4:** Parameters and statistics of the regression equations used for fitting the relationships between the qPCR estimation of the number of *Plasmopara viticola* oospores per gram of leaf (dependent variable Y) and 1) the microscope counts of oospores (independent variable X) and 2) quantification cycle (Cq, independent variable X).

Equation^a^	Equation parameters			
	a	b	c	R^2 c^
(1) log_10_(est) = a + b×log_10_(obs)	−0.147 ± 0.362^b^	1.157 ± 0.100	–	0.70
(2) log_10_(est) = c/(1+exp(−a + b×Cq))	15.285 ± 1.790	0.497 ± 0.060	3.828 ± 0.076	0.87

^a^Equation 1 is a linear regression Y = a+bX: y refers to the log_10_ transformed qPCR estimation (est) of the number of oospores per gram of leaf, and x refers to the log_10_ transformed of observed (obs) number of oospores in microscope counts; Equation 2 is a logistic equation Y=c/(1+exp(−a+bX)): y refers to the log_10_ transformed qPCR estimation (est) of the number of oospores per gram of leaf, and x refers to the Cq value. ^b^Standard errors of the estimated parameters. ^c^Coefficient of determination.

## Discussion

In 2005, Valsesia et al. (2005) described a qPCR assay for the detection of *P. viticola* in fresh grape leaves. The current study assessed the suitability of that assay (with minor modifications) for the quantification of pathogen’s overwintering inoculum, i.e., oospores in senescent grapevine leaves. One expectation in developing a detection method is that the new method performs better than or at least as well as an existing one. In our experiments, the qPCR assay was therefore compared with microscope counts of oospores. Our results showed that the two methods are comparable in that there was a strong linear relationship between the number of *P. viticola* oospores per gram of grapevine leaves estimated by qPCR and enumerated with the microscope. Furthermore, unlike microscope counts, the qPCR assay could detect significant differences among samples with low vs. no oospore infestation (25 vs. 0% infestation level), and was not influenced by the leaf sample weight, indicating that the qPCR method provides a more sensitive and reliable estimate of the number of oospores than microscope counts. In the comparison of the two methods (qPCR and microscope counts), it is also important to note that the microscopic counting of oospores in leaf tissue or in suspensions requires careful observation by experts. In our experiments, for example, the *P. viticola* oospores in suspensions could be easily confused with similar structures visible at the same magnification (e.g., spores of soilborne pathogens and saprophytes). Moreover, despite the thorough and time-consuming filtration process used to prepare oospore suspensions, oospores could remain trapped in microscopic fragments of leaf tissue, making observation and identification difficult. The qPCR assay, in contrast, provides less subjective estimates of *P. viticola* oospore numbers. These results, which need to be confirmed with a range of field samples, show that the qPCR assay provides accurate and consistent results.

It can be argued that the qPCR estimation is based on the quantification of total genomic DNA of *P. viticola* in leaf samples, which may include the oospores but also the endophytic mycelium that generated the oospores (Lafon and Clerjeau, 1988; Gobbin et al., 2006; Rumbou and Gessler, 2006), which would result in an overestimation of the number of oospores. The amount of mycelium in the leaf tissue, however, should be proportional to the number of oospores because the oospores form following crosses of antheridia and oogonia produced by the mycelium itself (Wong et al., 2001; Scherer and Gisi, 2006); therefore, if an overestimation exists, it should be proportional to the oospore numbers. Consistent with the latter assumption, the results of the present work revealed a direct, linear relationship between the quantity of *P. viticola* DNA detected by qPCR and microscope counts in senescent grapevine leaves that would soon drop to the soil surface and become leaf litter.

Both methods (qPCR and microscope counts) fail to distinguish between living and dead oospores, and that could bias the assessment of the inoculum potential in a vineyard. Long-term survival of oospores is influenced by several factors. For instance, exposure to high temperatures (40 to 53°C) for 1 to 24 h resulted in the death of oospores of *Phytophthora capsici* (Etxeberria et al., 2011) and the inhibition of the germination of oospores of *P. kernoviae* and *P. infestans* (Fay and Fry, 1997; Widmer, 2011). *Trichoderma asperellum*, a common soil-borne fungus, can penetrate oospores of *P. capsici*, develop hyphae, and produce conidia leading to the disintegration of oospores (Jiang et al., 2016). Other microorganisms, including the biocontrol agents *Bacillus subtilis* and *Trichoderma hartianum* T39, can prevent the germination of *Plasmopara viticola* oospores (Vecchione et al., 2005; Dagostin et al., 2006). Glucosinolates and their degradation products, which are generated when *Brassica* plants are incorporated into soil as green manure, also prevented the germination of *Pythium irregulare* oospores (Manici et al., 2000) and significantly reduced the viability of oospores of *P. capsici* when used in combination with solarization (Lacasa et al., 2015). Therefore, the quantification of total oospores (viable and non-viable) may overestimate the inoculum potential in a vineyard. Methods to differentiate between dead and viable cells by quantifying only DNA from viable cells have been developed (Fittipaldi et al., 2012); when propidium monoazide (PMA) is used with qPCR, for example, the PMA enters non-viable cells, binds to DNA, and inhibits DNA amplification during PCR (Nocker et al., 2007). A method that used PMA-qPCR to quantify the viable resting spores of *Plasmodiophora brassicae* in soil was developed by Al-Daoud et al. (2017). This method was subsequently used to demonstrate that a large proportion of the DNA of *P. brassicae* detected in soil was derived from non-viable or immature resting spores (Gossen et al., 2019). Adaptation of the method of Al-Daoud et al. (2017) for *P. viticola* would probably result in a more accurate and reliable estimation of the inoculum density, and this warrants further study.

In this work, the qPCR assay was used to detect the oospores in grapevine leaves, but oospores in grapevine leaves represent only part of the total oospores present in a vineyard. Once the leaf litter decomposes, the oospores are incorporated into the soil. Although these gradually die, some remain viable for at least 65 months (Caffi et al., 2011). The oospore population in a vineyard is therefore composed of oospores in the leaf litter and in the soil. There is no information about the epidemiological role of the oospores in the soil and their ability to produce zoospores that can be splash-transported from soil to grape leaves, but it would be useful to determine whether the qPCR assay can be used to estimate oospore numbers in vineyard soil samples. In this regard, researchers have recently developed techniques that overcome some of the problems in using qPCR to quantify pathogen inoculum in soil (Pavón et al., 2008; Schena et al., 2013; Hussain et al., 2014; Gossen et al., 2019; Van der Heyden et al., 2019). Another limitation of this research is that it was conducted with diseased senescent leaves that would have soon fallen to the soil surface and become litter rather than with leaf litter. It follows that the qPCR method and microscopic counts should now be compared for determination of oospore numbers in grapevine leaf litter.

The qPCR assay for *P. viticola* oospores could be useful for managing DM in vineyards. Such management is currently based on the assumption that the potential for severe disease outbreak is always present, even when the disease was not severe in the previous season. With this assumption, vineyard managers tend to apply fungicides whether they are needed or not. Managers would be less likely to apply unneeded fungicides if they knew the oospore inoculum levels (as indicated by the qPCR assay) in their vineyards and how those levels related to DM severity. The modification of disease management according to inoculum level has occurred with other pathosystems. Apple scab, for example, can be effectively controlled by delaying fungicide applications based on estimates of the potential ascospore numbers of *Venturia inaequalis* in an orchard (MacHardy et al., 1993). Information on the *P. viticola* oospore numbers in vineyards could also be used for adapting the mathematical models for predicting *P. viticola* oospore dynamics and primary infections (Caffi et al., 2007; Rossi et al., 2008b; Caffi et al., 2009). Further study could be performed in order to establish the relationship between oospore density in vineyard and DM severity.

## Data Availability Statement

The raw data supporting the conclusions of this article will be made available by the authors, without undue reservation.

## Author Contributions

VR, MS, and ST mainly contributed to the conception and the design of the study. MS and FB carried out the experiments. VR, MS, and FB contributed to the analysis of results. MS and FB wrote the first draft of the manuscript. VR and ST contributed to the critical analysis of the manuscript. All authors contributed to the article and approved the submitted version.

## Funding

Financial support for carrying out this research was partially provided by the Project BIOVINE supported by transnational funding Bodies, being partners of the H2020 ERA-net project "CORE Organic Cofund", and the cofund from the European Commission.

## Conflict of Interest

The authors declare that the research was conducted in the absence of any commercial or financial relationships that could be construed as a potential conflict of interest.
